# Extent and Correlates of Self-Medication Practice among Community-Dwelling Adults in Eastern Ethiopia

**DOI:** 10.1155/2023/4726010

**Published:** 2023-11-30

**Authors:** Abdu Oumer, Ahmed Ale, Aragaw Hamza, Imam Dagne

**Affiliations:** ^1^Department of Public Health, College of Medicine and Health Science, Dire Dawa University, Dire Dawa, Ethiopia; ^2^School of Medicine, College of Medicine and Health Science, Dire Dawa University, Dire Dawa, Ethiopia; ^3^Department of Anesthesia, College of Medicine and Health Science, Dire Dawa University, Dire Dawa, Ethiopia

## Abstract

**Background:**

The use of medications without proper medical consultations poses significant health risks, drug resistance, and undiagnosed disease conditions, becoming a major pharmaceutical challenge in the 21^st^ century. This study assessed the magnitude and associated factors of self-medication practice among adults in parts of Ethiopia.

**Methods:**

A community-based cross-sectional study was conducted among 647 randomly selected adults residing in randomly selected households in eastern Ethiopia via a stratified sampling approach. A pretested interviewer-administered questionnaire was used to collect the data on self-medication practice. Data were presented using tables, frequencies, percentages, and graphs. A multivariable binary logistic regression was done to identify factors associated with self-medication practice and presented as an adjusted odds ratio along with its 95% CI. Associations with a *p* value below 5% were used to declare statistical significance.

**Results:**

A total of 647 adults with a mean age of 41.7 (11.4) years were included. Overall, 15.8% (95% CI: 12.5–18.2) of them reported to have practiced self-medication in the past month, while 67.9% (95% CI: 64.1–74.7) have practiced self-medication, mainly due to the mild nature of the symptom (11%), intention to get a rapid cure (12.2%), physical accessibility (9.1%), and less confidence in the quality of health facility services (3.7%). The majority of the drugs were in the form of oral tablets in the antibiotic, antipain, and gastrointestinal categories. Female (AOR = 1.66 and 95% CI: 0.76–3.61), larger family size (AOR = 1.34 and 95% CI: 0.73–2.46), illiteracy (AOR = 4.47 and 95% CI: 1.17–17.1), poor socioeconomic class (AOR = 4.6795 and CI: 1.71–12.7), perceived health facility visit stay as long (AOR = 1.55 and 95% CI: 0.80–3.00), khat use (AOR = 2.86 and 95% CI: 1.27–6.47), cigarette smoking (AOR = 2.86 95% CI: 1.27–6.47), and poor knowledge on proper medication use (AOR = 7.98 and 95% CI: 4.61–13.8) were associated with increased odds of self-medication.

**Conclusion:**

The practice of self-medication is a health concern and is associated with lower socioeconomic class, illiteracy, substance abuse, a perceived long stay at a health facility, and poor knowledge of medication use. Behavioral interventions targeting this segment of the population via various approaches would help.

## 1. Introduction

Self-medication can be defined as the self-consumption of medication without getting advice from a physician for either diagnosis or treatment. However, the risks and accessibility of medicine for self-treatment may vary depending on the regulations. Medicines can be in the categories of over-the-counter (OTC) drugs and prescription-only medicines (POM). OTC drugs are those that can be dispensed and consumed without medical supervision [1, 2]. Self-medication also includes acquiring medicines without a prescription, resubmitting old prescriptions to purchase medicines, sharing medicines with relatives or members of one's social circle, or using left-over medicines stored at home [3–5].

Self-medication can readily relieve acute medical problems, save time spent waiting to see a doctor, be economical, and even save lives in acute conditions [5–7]. In many cases, self-medication can be an important initial response to illness or symptoms [1, 5]. In most economically poor countries, most episodes of illness are treated by self-medication, and many drugs are dispensed as OTC without medical supervision. In this case, self-medication provides a lower-cost alternative for patients who cannot afford the cost of clinical services [8]. However, this practice could pose a significant challenge for POMs. The use of self-prescribed medication and left overs is on the rise. Self-treatment has the potential to lead to several health problems, including the misuse of OTC medication, the concurrent use of several medications, and the use of home remedies to treat potentially serious diseases. This can potentially result in misdiagnosis or the masking of potential health problems [9, 10].

Self-medication is one of the most frequently observed irrational utilizations of medicines, both in developed and developing countries, that leads to bad consequences from drugs. Self-medication will result in the wastage of resources, increase the resistance of pathogens, and generally lead to serious health hazards and prolonged suffering [11]. Today, the use of POM drugs without professional advice is becoming more common around the world. However, the practice will have so many consequences, like bacterial resistance, drug interactions, a prolonged treatment period, serious side effects, failure to achieve optimal treatment, intentional and unintentional poisoning, an increase in malignant and lethal diseases, and drug dependence [5]. The use of medicines without the help of health professionals will lead to serious problems, including the global emergence of multidrug resistance pathogens, drug dependence, addiction, masking of malignant and potentially fatal diseases, hazard of misdiagnosis of problem, relation to over- and underdosage, drug interaction, and tragedies relating to the side effect profile of specific drugs [12].

The prevalence of self-medication practice was 65.8%, and traditional medicines are more practiced than modern medicine [13]. A study done in Ethiopia found that the prevalence of self-medication was 27.5% in a two-week recall period. From these, 13.8% of the self-medicated people used modern drugs [14]. Clients usually prefer to manage their common health problems using self-medication, as it is easier, more cost-effective, and more time-efficient. However, it is associated with many adverse health consequences [15]. Considering the prevailing self-medication practice in the current poorly regulated regulatory systems, the availability of drugs in nearby drug shops, the conducive drug market, the improvement of people's awareness, and other conditions, it is necessary to study the level of self-medication practice and factors leading to self-medication in these near-border cities of the country. Moreover, the recent COVID-19 pandemic restriction had a significant impact on medication access and access to health services [16–18], which could affect the practice of self-medication. It will be informative on the existing self-medication practices in the region in order to take appropriate measures.

## 2. Materials and Methods

### 2.1. Study Settings

This study was conducted in the Dire Dawa city administration and Harar town, in the eastern parts of Ethiopia. Harar is located 526 kilometers from Addis Ababa, the capital city of Ethiopia. Based on the 2007 Ethiopian Central Statistics Authority's population projection, there are 250,093 people, with 146,913 living in urban areas and 122,942 being male, for a total household size of 64,334. Dire Dawa has an estimated population of 506,936 as of 2012 EFY, with an estimated 76,000 households. In Dire Dawa, there are two public hospitals. This study was conducted from May to June 2022 for more than one month.

### 2.2. Study Design and Population

A community-based cross-sectional study design was conducted to elicit self-medication practice and its associated factors among randomly selected adult community dwellers aged above 18 living in Dire Dawa and Harar, eastern Ethiopia. The study did not include households with adults who are seriously ill and unable to communicate or adults who have no history of illness in the past month.

### 2.3. Sample Size Determination and Sampling Procedures

The minimum sample size was estimated using different scenarios. First, we employed a single-proportion sample size formula with “*P*” as the prevalence of self-medication practice among adults from the previous study (*P*_1_ = 24.4% and *n*_1_ = 283 [19], *P*_2_ = 36.8% and *n*_2_ = 357 [20], and *P*_3_ = 52.5% and *n*_3_ = 383 [21]), 95% confidence level, “*Z*” critical value at 95% confidence level (1.96), and marginal error “*d*” of 4%. The largest sample size for the first objective was 383. Secondly, the sample size for identifying the potential risk factors for self-medication using the StatCalc module in Epi Info, considering sex (AOR = 3.11), was estimated as 420. Hence, we chose the larger sample size for the second objective (424), and with a design effect of 1.5 and a 5% nonresponse rate, the final sample became 668.

A stratified random sampling with proportional allocation method was used to proportionally allocate the sample to the two study sites and then to the subsequent sampling units. Then, a random sample of woreda (4 districts) and kebeles (5 kebeles) was selected using simple random sampling for Harar and Dire Dawa, respectively. Sample households were selected from the randomly selected kebeles using systematic random sampling at every sampling fraction (*K*). The sample fraction was calculated for each kebele by dividing the total households in the kebele by the allocated sample size for that kebele (*K*_*i*_ = *N*_*i*_/*n*_*i*_). Then, after taking a random starting sample, households at each sampling interval (selected adults were interviewed) were included.

When households are closed during data collection, a consecutive revisit is scheduled, and if the household is still not accessible, it is skipped. While there is more than one eligible household member, preference was given to the household head or housewife. When these are not accessible, one adult is selected using random sampling and included in the study.

### 2.4. Methods of Data Collection

The data were collected using a set of structured questionnaires. Data were collected by trained health care workers or graduating health students through house-to-house face-to-face interviews. A structured interviewer-administered questionnaire was used. The questionnaire is prepared from a literature review of previous studies conducted in different areas. The questionnaire includes sociodemographic information, the practice of self-medication and reasons for its use, and knowledge about self-medication. The self-medication practice of respondents was elicited with the recent one-month recall period with the aim of capturing the true magnitude of the problem, as a shorter recall period may underestimate its magnitude.

### 2.5. Data Quality Assurance

Data collectors were trained for about two days on the tool and how to elicit self-medication practices. Constructive feedback was given to the data collectors by the investigators and supervisors to make them familiar with the tool and approach while training and pretesting the tool and setting. During data entry into EpiData, the data quality was maintained by making legal ranges, skipping patterns, appropriate coding, and careful data entry. The tool was pretested in 20 households in a nonselected kebele in Harar to evaluate its suitability, and necessary amendments were made accordingly. In addition, the clarity, completeness, and relevance of the questions used in the tool were thoroughly checked. The data were entered in a controlled data entry format and checked for consistency, and a clean copy of the data was used for analysis.

### 2.6. Methods of Data Analysis

After checking for completeness and inconsistencies, the data were entered into EpiData version 3.02 and exported to SPSS version 20 for analysis. The data were presented in tables, graphs, percentages, frequencies, means, medians, and standard deviations. The outcome variable of self-medication practice was coded as 1 (yes) or 0 (no) and summarized in tables or figures. A bivariable and multivariable binary logistic regression analysis was done to assess the factors associated with self-medication practice. Variables with a *p* value below 0.25 and other important factors were included in the multivariable binary logistic regression [22, 23]. The crude and adjusted odds ratios (COR and AOR) with their 95% confidence intervals were used and reported to assess the strength of the association between factors and self-medication practice. The omnibus test with its *p* value was used to assess the effects of variables on the model specification. Hosmer and Lemeshow's goodness-of-fit measure was used to assess the model's fitness, and a *p* value association with a *p* value below 0.05 was used to declare a statistically significant association.

### 2.7. Variables

The dependent variable of this study was self-medication practice, and the independent variables were demographic variables (age, sex, income, occupation, age, marital status, educational status, and income), perceived availability and quality of health care, having a health professional friend or family, peer/family influence, knowledge, lack of sufficient time, perception on quality, cost of health care, and access to pharmacy/drug shop.

### 2.8. Ethical Considerations

Ethical review and support letter was obtained from the Dire Dawa University, Institutional Review Board, Research Ethics Review Committee. Verbal informed consent was obtained from each respondent after explaining the details of the study procedure. The data is going to be used for this research only. Personal identification of clients like name, place, and other sensitive information were not recorded. Hardcopy and softcopy data will not be disclosed to a third party without their full consent.

## 3. Results

### 3.1. Sociodemographic Characteristics

In this study, a total of 647 adults participated, with a response rate of 97%. More than two-thirds of the respondents (66.9%) were female, with an overall mean age of 41.7 (11.4) years. About 495 (76.5%), 278 (43%), and 215 (33.2%) of them were married, attended college or above, and worked in government institutions, respectively. While 260 (40%) of them were from poor households, a total of 248 (38.4%) of the respondents belong to wealthier households based on household asset measurements. About 399 (61.7%) of the respondents were from households where the family size was below five **(**Table 1).

Moreover, 237 (36.6%) of the respondents had health insurance packages for themselves and/or their families. In this study, 120 (18.5%) and 63 (9.7%) of the respondents reported regularly drinking alcohol and smoking cigarettes, respectively. Moreover, khat consumption was found to be prevalent, with more than three-fourths (80.5%) of them having a habit of khat chewing.

**Figure 1:**
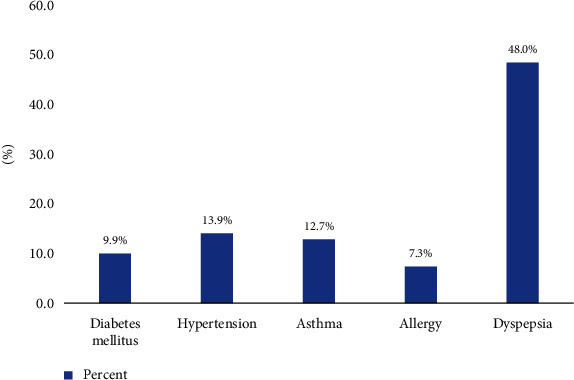
Prevalence of major morbidities among adults residing in the eastern part of Ethiopia, 2022.

In the current study, 90 (13.9%), 82 (12.7%), and 47(7.3%) of adults reported to have hypertension, asthma, and allergy, respectively. In addition, dyspepsia was found to be the common comorbid condition which is reported by 48.8% of the respondents (Figure 1). Moreover, a total of 119 (18.4%) rated that the stay at a health facility is very long (at least two hours).

### 3.2. Knowledge on Self-Medication

The knowledge of study participants about self-medication was assessed using nine-item questions. The detailed responses and level of understanding of the study participants are presented in Table 2. The overall knowledge score of the study participants was 6.5 (±1.9) out of a total score of nine. Based on a cut-off point of below seven, 194 (30%) of them reported having poor knowledge of proper medication use (Table 2).

### 3.3. Magnitude of Self-Medication Practice

Over the past month, a total of 438 (67.7%) reported having some sort of illness. Among these, 359 (55.5%) decided to visit a health facility, while 60 (9.3%) took medicine by themselves. Among those who practiced self-medication over the past month, 12.1%, 5.9%, and 4.3% of them used the drugs to treat gastrointestinal symptoms, cold and/or cough, and fever/headache syndromes, respectively. On average, they reported taking one to five drugs, while the majority took less than three drugs. Gastrointestinal drugs, antacids, and antibiotics were commonly consumed mainly in the form of oral tablets (Table 3).

Concerning the long-term prevalence of self-medication, 439 (67.9% and 95% CI: 64.1–74.7) reported taking medication without a prescription or consultation. The recent prevalence of self-medication was 98 (15.8% and 95% CI: 12.5–18.2) over the last one month. The commonly mentioned reasons for self-medication were the mild nature of the symptom (11%), the intention to get a rapid cure (12.2%), physical accessibility (9.1%), and a lack of confidence in the quality of health facility services (3.7%). Moreover, saving time and money due to the costly nature of facility care was also mentioned as a barrier (Table 3).

**Figure 2:**
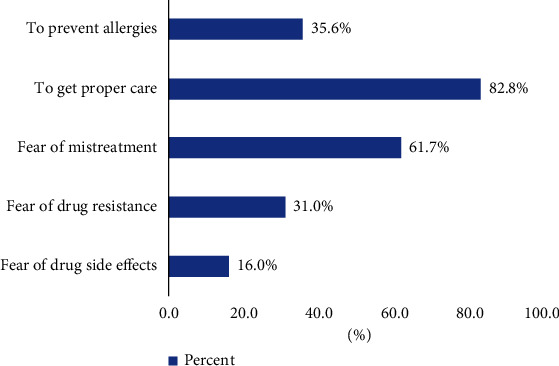
Mentioned reasons for not practicing self-medication among adults residing in eastern Ethiopia.

As indicated in Figure 2, the reason for not using self-prescribed medication was elicited. Among the common reasons, people tend to avoid self-medication to get proper health care service (82.8%); also, fear of mistreatment (over or underdose treatment) (61.7%), fear of drug resistance (31%), and potential drug side effects (16%) were the commonly reported barriers to self-medication practices (reasons for not practicing self-medication among adults residing in eastern Ethiopia are listed in Figure 2).

### 3.4. Factors Associated with Self-Medication among Adults

A stepwise binary logistic regression was carried out to identify factors associated with self-medication practice. For the purpose of getting a recent and more reliable (with minimum recall bias) prevalence estimate, we considered the prevalence of self-medication for the past one-month period. Overall, marital status, educational status, wealth index, knowledge of proper medication use, and substance use were found to be significantly associated with self-medication practices among adults. Hence, self-medication practice was more prevalent among females (COR = 1.63 and 95% CI: 0.99–2.67), currently unmarried (COR = 1.96 and 95% CI: 1.06–3.63), lower wealth quintiles (COR = 2.79 and 95% CI: 1.32–5.92), and illiterates (COR = 6.49 and 95% CI: 2.41–17.5) compared to their counterparts. Similar patterns were observed among those who smoke cigarettes (*p* value = 0.046), chew khat (*p* value = 0.006), and had poor knowledge of proper drug uses (*p* value = 0.0001) (Table 4).

A multivariable regression model was fitted using a backward stepwise regression method considering the model stability, *p* value, and/or biological plausibility of the factors. Overall, Hosmer and Lemeshow's model fitness value for this study was 0.378, indicating a fit model. The result showed that females (AOR = 1.66 and 95% CI: 0.76–3.61), larger family size (AOR = 1.34 and 95% CI: 0.73–2.46), and currently unmarried respondents practice self-medication more often compared to their counterparts. Surprisingly, self-medication was common among the illiterate (AOR = 4.47 and 95% CI: 1.17–17.1) and primary school students (AOR = 1.55 and 95% CI: 0.66–3.67) as compared to those who attended college and above. Self-medication practice was found to be common among the poor, which is statistically significant (*p* value below 0.05). Interestingly, those participants who perceived the health facility visit stay as long (AOR = 1.55 and 95% CI: 0.80–3.00) had higher odds of taking medication without a prescription. Practicing substance use, specifically khat use (AOR = 2.86 and 95% CI: 1.27–6.47) and cigarette smoking (AOR = 2.86 and 95% CI: 1.27–6.47), was associated with 2.8- and 4.2-times increased odds of taking self-prescribed medications, respectively. Having poor knowledge of self-medication was strongly and significantly associated with practicing self-medication (AOR = 7.98 and 95% CI: 4.61–13.8) (Table 5).

## 4. Discussions

Currently, the occurrence of mistreatment and associated drug-related problems is creating a global challenge [4]. These are increasing the incidence of drug side effects and drug resistance, delaying the diagnosis and identification of clinically relevant illnesses, hence delaying appropriate care [6]. These would increase the disease burden due to morbidity and preventable mortality in the population [7]. Hence, it is imperative to understand the magnitude of self-medication in certain local contexts, which helps identify the potentially preventable risk factors for targeted intervention on proper medication use [24]. Although self-medication with potentially hazardous medication might create a serious public health concern for customers, the potential advantages of self-medication in some particular cases are beneficial [4, 25]. Hence, it is crucial to limit the extent of self-medication regulatory systems with a focus on non-OTC drugs [5].

The findings of this study showed that 15.8% of the respondents practiced self-medication over the past month, while 69.7% have practiced self-medication. This indicates that the practice of self-medication is more common in the study area, where the majority of the drugs were PODs. In comparison to another study done in Uganda, where 75.7% of adults practiced antimicrobial self-medication in the treatment of fever, headache, lack of appetite, and body weakness, surprisingly, 46.5% and 57.6% were self-initiated and recommended, respectively, by drug shop attendant respondents [26]. Evidence from Togo for COVID-19 cases showed a higher medication use rate of 34.2% [27]. A various level of self-medication was reported in Ethiopia. For instance, community-level self-medication was 35.9% [14], with 32.6% from northwest Ethiopia [13], and 35% of respondents had self-medication histories [14]. In our study, the recent self-medication history was comparably low, but a comparable self-medication occurrence has been reported. The practice of self-medication was consistent in terms of the common symptoms to treat fever, headache, pain, and gastrointestinal symptoms. For example, another study showed that fever (31%), abdominal pain (16.7%), and headache were the most common reasons to take medications [28]. This has been further aggravated by the recent COVID-19 pandemic [18, 29, 30].

People react differently when they encounter an illness where the selected treatment strongly depends on socioeconomic and cultural factors [24]. Our finding showed that being from lower socioeconomic classes and having lower educational status are significantly associated with higher odds of practicing self-medication compared to counterparts. This could be related to multiple dimensions. For instance, other segments of the population could have better knowledge and economic access to appropriate medical care at government and/or private health facilities. This is supported by knowledge that proper medical care is associated with low self-medication practice. These findings are supported by one study which indicated that, in addition, families with a higher educational status were less likely to store and share medicines which is positively associated with self-medication [31].

Substance users, specifically those who consume khat and smoke cigarettes, are associated with higher odds of self-medication. Another study has shown that substance use, such as drinking alcohol (AOR = 1.84; 1.21–2.80) and khat use (AOR = 1.03; 0.87–1.84), is associated with a higher risk of self-medication [32]. This might be associated with the occurrence of more frequent substance use-related illnesses and addictions, which might increase self-medication practices [33]. However, the case of alcohol was not depicted in our study.

Knowledge of proper medication use is an important determinant of self-medication, which might partly be explained by being from a poor socioeconomic class or having a lower educational status. A similar study among adolescents showed that those with better medication knowledge (AOR = 0.42; 0.22–0.81) and medication literacy (AOR = 0.97; 0.93–1.08) were less likely to engage in inappropriate self-medication practices [32].

Improper care at health facilities, like a longer stay at the facility and perceived poor service quality, could convince clients to go for more user-friendly self-medication practices. This is also clearly indicated in our study, where a perceived long stay at a health facility (AOR = 1.55 and 95% CI: 0.80–3.00) had 55% more odds of taking medication without a prescription. It would be imperative to strengthen measures to increase the quality of care at health facilities by increasing access and reducing improper self-medication practices. Other studies have also shown that those who perceived poor service quality at health facilities (AOR = 4.67; 2.56–7.96) and easily accessed pharmacies or drug shops (AOR = 2.32; 1.65–6.76) were more likely to take medicine without a prescription [21]. Other studies have also shown similar results [19, 31, 34, 35]. Hence, decreased physical accessibility and the inability to get medication with ease due to poor regulatory mechanisms are encouraging customers to go for self-treatment [36, 37]. The implementation of regulatory systems concerning the marketing of prescription-only drugs with potential harm to the user or the community should be strengthened [1, 7, 19, 31, 33–35, 37].

Due to the survey nature of the study and some challenges in having a self-medication practice, the findings of this study will be limited [38]. Although the risk of recall bias might be low in our case due to the shorter recall period (one month), the respondents might still underestimate the medication type, dose, form, and other information. Hence, the finding might be limited in generalizability, and the study findings might not be representative of eastern Ethiopia.

## 5. Conclusion and Recommendations

Generally, the prevalence of self-medication in the study area is relatively higher where the majority of the drugs are non-OTC drugs. The practice of self-medication is associated with lower socioeconomic class, illiteracy, substance use, perceived long health facility visits, and poor knowledge of medication use. Strong behavioral interventions with a focus on these segments of the population through various approaches should be in place. The regional health bureaus, through strong regulatory frameworks, should strengthen regular monitoring and supervision. Drug shop and/or pharmacy workers and owners should strongly advise customers to go for proper medical care and avoid unnecessary self-medication practices. Health facilities should take appropriate measures to increase service quality and shorten stays at facilities to reduce associated costs for clients.

## Figures and Tables

**Table 1:** Sociodemographic characteristics of adult study participants for the self-medication study in eastern Ethiopia, 2022.

Variables	Categories	Frequency (*n*)	Percent (%)
Sex of the respondent	Female	433	66.9
	Male	214	33.1

Marital status	Married	495	76.5
	Single	73	11.3
	Widowed	45	7.0
	Divorced	34	5.3

College and above	Illiterate	19	2.9
	Primary school	170	26.3
	Secondary school	180	27.8
	College and above	278	43.0

Occupation	Government	215	33.2
	Housewife/worker	195	30.1
	Private worker	159	24.6
	Do not have work	70	10.8
	Farmer	8	1.2

Family size	≤4	399	61.7
	>4	248	38.3

Wealth index	Poorest	131	20.2
	Poorer	129	19.9
	Middle	139	21.5
	Wealthier	113	17.5
	Wealthiest	135	20.9

Age of the participant (mean ± sd)	41.7 (11.4) years		
Family size (mean ± sd)	4 (1.5)		

**Table 2:** Assessment of the knowledge and perception of adults towards self-medication practice in eastern Ethiopia, 2022.

Variables		Frequency (*n*)	Percent (%)
Self-medication could result in wastage of money	No	97	15.0
	Yes	550	85.0
Self-medication could result in drug resistance	No	102	15.8
	Yes	545	84.2
Self-medication could result in masking symptoms of underlying disease	No	121	18.7
	Yes	526	81.3
Do you think that medicine should be sold like any other goods?	Yes	47	7.3
	No	600	92.7
Do you perceive that any drug can cure any disease?	Yes	16	2.5
	No	631	97.5
Did you ever check the instructions that come with the package of the drug for self-treatment?	No	274	42.3
	Yes	373	57.7
Do you fully understand the information or the instruction in the package?	No	332	51.3
	Yes	315	48.7
Were you concerned about taking counter-opposing medicines?	No	483	74.7
	Yes	164	25.3
Is self-treatment/self-medication a good practice?	Yes	149	23.0
	No	498	77.0
Knowledge level towards self-medication	Poor knowledge	194	30.0
	Good knowledge	453	70.0
Knowledge score (mean (±sd))	6.5 (±1.9)		

**Table 3:** Reported illness history and magnitude of self-medication practice among community-dwelling adults in Ethiopia.

Items	Options	Frequency (*n*)	Percent (%)
Illness in the past one month	No	209	32.3
	Yes	438	67.7

What was your measure (*n* = 438)	Visit health facility	359	55.5
	Take medicine by my self	60	9.3
	Seek recovery	1	.2
	Consult health professionals personally	18	2.8

Self-medication in the last month	No	549	84.9
	Yes	98	15.1

Self-medication was for	Fever/headache	28	4.3
	Cold and cough	38	5.9
	Gastrointestinal symptoms	78	12.1
	Back pain	26	4.0
	Dysmenorrhea	8	1.2

Number of drugs used	1	17	2.6
	2	51	7.9
	3	29	4.5
	5	1	.2

Type of drug used	Over-the-counter drugs	17	17.2
	Prescription-only drugs	81	80.7

Forms of drug used	Injections	2	0.3
	Oral tablets	95	14.7

Group of the drug(s) used	Antibiotics	38	5.9
	Antipain	72	11.1
	GI drugs	84	13.0
	Antacids and others	14	2.2%

How do you identify the doses (*n* = 98)	By checking the package	2	.3
	By consulting family members/friends	23	3.6
	Consulting doctors/health professionals	16	2.5
	Previous experience	49	7.6

Who advised you to take medication (*n* = 96)	Friends	18	2.8
	Professionals	21	3.2
	Myself	56	8.7
	Neighbors	2	.3

Self-medication history before the current illness	No	254	39.3
	Yes	393	60.7

Have you ever treated yourself with self-medication without prescription	No	208	32.1
	Yes	439	67.9

Reason for choosing self-medication	Being mild illness	71	11.0
	To get rapid cure	79	12.2
	To save time and money	13	2.0
	Physically accessible	59	9.1
	Not confident in health facility services	24	3.7
	Peer influence	5	0.8

**Table 4:** Bivariable logistic regression showing the factors associated with self-medication practice among adults in eastern parts of Ethiopia.

Factors	Categories	Self-medication		COR (95% CI)	*p* value
		Yes	No		
Sex	Male	24	190	1	
	Female	74	359	1.63 (0.99-2.67)	0.051

Age in years	20-34	23	125	1	
	35-50	32	200	0.87 (0.48-1.55)	0.637
	≥50	43	224	1.04 (0.60-1.81)	0.880

Marital status	Married	62	433	1	
	Single	16	57	1.96 (1.06-3.63)	0.032^∗^
	Widowed	10	35	2.00 (0.94-4.23)	0.072
	Divorced	10	24	2.91 (1.33-6.38)	0.008^∗∗^

Family size	≤4	53	346	1	
	>4	45	203	1.45 (0.94-2.23)	0.095

Educational status	Illiterate	8	11	6.49 (2.41-17.5)	0.0001^∗∗^
	Primary school	31	139	1.99 (1.15-3.46)	0.014
	Secondary school	31	149	1.86 (1.07-3.21)	0.027^∗^
	College and above	28	250	1	

Occupational status	Government	15	200	1	0.0001^∗∗^
	Nongovernmental worker	82	349	3.17 (1.78-5.65)	

Wealth index	Poorest	26	105	2.79 (1.32-5.92)	0.007^∗∗^
	Poorer	19	110	1.95 (0.89-4.27)	0.096
	Middle	23	116	2.24 (1.04-4.79)	0.039^∗^
	Wealthier	19	94	2.28 (1.04-5.02)	0.041^∗^
	Wealthiest	11	124	1	

Cigarette smoking	No	83	501	1	
	Yes	15	48	1.89 (1.01-3.52)	0.046^∗^

Drink alcohol	No	83	444	1.31 (0.73-2.36)	0.371
	Yes	15	105	1	

Chew khat	No	9	119	1	
	Yes	89	430	2.74 (1.34-5.59)	0.006^∗∗^

Stay at health facility	Long (≥2 hours)	23	96	1.45 (0.86-2.43)	0.161
	Short (<2 hours)	75	453	1	

Knowledge on self-medication	Poor	68	126	7.61 (4.74-12.2)	0.0001^∗∗^
	Good	30	423	1	

Note: associations are statistically significant at a *p* value below 0.05 (^∗^) and *p* value below 0.01 (^∗∗^).

**Table 5:** Multivariable logistic regression output showing the factors associated with self-medication practice among adults in eastern parts of Ethiopia, with AOR and its corresponding *p* value reported.

Factors	Categories	Self-medication		AOR (95% CI)	*p* value
		Yes	No		
Sex	Male	24	190	1	
	Female	74	359	1.66 (0.76-3.61)	0.201

Family size	≤4	53	346	1	0.35
	>4	45	203	1.34 (0.73-2.46)	

Marital status	Married	62	433	1	
	Single	16	57	2.85 (1.12-7.23)	0.028^∗^
	Widowed	10	35	1.41 (0.58-3.41)	0.444
	Divorced	10	24	2.79 (1.07-7.25)	0.035^∗∗^

Educational status	Illiterate	8	11	4.47 (1.17-17.1)	0.028^∗^
	Primary school	31	139	1.55 (0.66-3.67)	0.315
	Secondary school	31	149	2.61 (1.21-5.66)	0.015^∗^
	College and above	28	250	1	

Wealth index	Poorest	26	105	4.67 (1.71-12.7)	0.003^∗∗^
	Poorer	19	110	5.35 (2.05-14.0)	0.001
	Middle	23	116	4.68 (1.94-11.28)	0.001^∗^
	Wealthier	19	94	2.52 (1.04-6.10)	0.040^∗^
	Wealthiest	11	124	1	

Cigarette smoking	No	83	501	1	
	Yes	15	48	4.21 (1.62-11.0)	0.003^∗∗^

Chew khat	No	9	119	1	
	Yes	89	430	2.86 (1.27-6.47)	0.012^∗^

Stay at health facility	Long (≥2 hours)	23	96	1.55 (0.80-3.00)	0.196
	Short (<2 hours)	75	453	1	

Knowledge on self-medication	Poor	68	126	7.98 (4.61-13.8)	0.0001^∗∗^
	Good	30	423	1	

Note: associations are statistically significant at a *p* value below 0.05 (^∗^) and *p* value below 0.01 (^∗∗^).

## Data Availability

All relevant data are within the manuscript and its supporting information files.

## Abbreviations

A/COR: Adjusted/crude odds ratio
CI: Confidence interval
GI: Gastrointestinal drugs
OTC: Over the counter
POM: Prescription-only medicine
WHO: World Health Organization.
